# Microbial Adhesion and Cytotoxicity of Heat-Polymerized and 3D-Printed Denture Base Materials when Modified with Dimethylaminohexadecyl Methacrylate and/or 2-Methacryloyloxyethyl Phosphorylcholine as Antimicrobial and Protein-Repellent Materials

**DOI:** 10.3390/polym17020228

**Published:** 2025-01-18

**Authors:** Njood F. AlAzzam, Salwa O. Bajunaid, Bashayer H. Baras, Heba A. Mitwalli, Michael D. Weir, Hockin H. K. Xu

**Affiliations:** 1 Department of Prosthetic Sciences, College of Dentistry, King Saud University, Riyadh 60169-15, Saudi Arabia; sbajunaid@ksu.edu.sa; 2 Department of Restorative Dental Sciences, College of Dentistry, King Saud University, Riyadh 60169-15, Saudi Arabiahmitwalli1@ksu.edu.sa (H.A.M.); 3 Department of Advanced Oral Sciences and Therapeutics, University of Maryland School of Dentistry, Baltimore, MD 21201, USA; michael.weir@umaryland.edu (M.D.W.); hxu2@umaryland.edu (H.H.K.X.)

**Keywords:** microbial adhesion, cytotoxicity, denture base, antimicrobial, protein-repellent

## Abstract

Background: Polymethyl methacrylate (PMMA) is ideal for denture bases but is prone to biofilm accumulation, leading to denture stomatitis (DS), often involving *Candida albicans*. Dimethylaminohexadecyl methacrylate (DMAHDM) and 2-methacryloyloxyethyl phosphorylcholine (MPC) are introduced into dental materials for their antimicrobial and protein-repellent properties. This study investigates the effects of incorporating dimethylaminohexadecyl methacrylate (DMAHDM) and 2-methacryloyloxyethyl phosphorylcholine (MPC) into heat-polymerized (HP) and 3D-printed (3DP) denture base resins on microbial adhesion and cytotoxicity. Methods: HP and 3DP denture base specimens were prepared using varying concentrations of DMAHDM and MPC. Microbial adhesion was quantified using CFU counts of *C. albicans*, and cytotoxicity was assessed via an MTT assay using fibroblast cells after 24 h, 3 days, and 7 days. Results: Both DMAHDM and MPC significantly reduced the CFU counts in both HP and 3DP materials; the combination of 1.5% DMAHDM and 3% MPC exhibited the most substantial antimicrobial effects. Cytotoxicity results varied between materials and time points; however, all treated groups maintained cell viability above the 70% threshold, indicating no significant cytotoxic effects. Conclusion: Incorporating DMAHDM and MPC into denture base resins can effectively reduce microbial adhesion while maintaining acceptable cytotoxicity levels.

## 1. Introduction

Polymethyl methacrylate (PMMA) acrylic resin is considered to be the material of choice for the fabrication of removable denture bases as it meets the majority of the criteria for the ideal denture base material [1,2]. Nevertheless, the major disadvantage of PMMA is its tendency to accumulate biofilm, as it acts as a pathogen reservoir and favors their colonization [2,3,4,5]. Mechanical attachment of the biofilm to the surface of PMMA may be influenced by certain local factors, such as surface porosities, roughness, poor oral or denture hygiene, and prolonged denture wear [6,7,8]. Recently, additive techniques were developed for the fabrication of removable denture acrylic bases and metal frameworks [9,10].

Denture stomatitis (DS) is an oral disease associated with various local and systemic factors, such as ill-fitting dentures, poor oral or denture hygiene, poorly fabricated prostheses, prolonged denture wear, xerostomia, and compromised immunity [11,12]. It is most commonly linked to the presence of *Candida albicans* on the denture’s fitting surface and the oral mucosa [13,14]. *Candida albicans* is a pathogen with low susceptibility to antifungal agents and significant resistance to antifungal therapy [15,16,17,18].

In 1993, the “immobilized bactericide” concept was introduced into the field of dental materials with an antibacterial monomer capable of co-polymerizing with methacrylate resin monomers [19]. Research has suggested that the greatest antimicrobial efficacy of dimethylaminohexadecyl methacrylate (DMAHDM) when incorporated into different dental materials has been found when the alkyl chain length (CL) of the ammonium group is increased to CL 16 [20,21,22]. 2-methacryloyloxyethyl phosphorylcholine (MPC) is a protein-repellent monomer that is capable of co-polymerizing with acrylic resin when using covalent bonding [23,24,25,26]. It is considered to be biocompatible and harmless to human tissues while also having a potential impact on reducing protein adsorption, cellular attachment, and microbial adhesion [27,28,29].

This study aims to examine the impact of incorporating DMAHDM and/or MPC into heat-polymerized and 3D-printed denture base resins on the accumulation and cytotoxicity of *C. albicans*. The hypothesis being tested was that incorporating DMAHDM and/or MPC into heat-polymerized or 3D-printed denture base resins would not affect the colony-forming unit (CFU) recovered from denture materials and/or the cytotoxicity of the developed material in comparison to the corresponding control groups lacking DMAHDM and/or MPC.

## 2. Materials and Methods

### 2.1. Incorporation of DMAHDM and MPC

DMAHDM was synthesized using a modified Menschutkin reaction method [30,31,32,33,34]. MPC was commercially purchased from Sigma-Aldrich (MPC; Sigma-Aldrich, St. Louis, MI, USA). Manual mixing of DMAHDM and MPC into the acrylic resin liquid was performed at mass fractions of 1.5% and 3% [34]. Acrylic resin liquid without MPC and/or DMAHDM (0%) served as the control group for comparison [30,31,34].

### 2.2. Preparation of the Testing Groups

#### 2.2.1. Heat-Polymerized (HP) Denture Base Material

The mixing of the heat-polymerized denture base material (ProBase Hot; Ivoclar Vivadent Inc., Mississauga, ON, Canada) was performed following the manufacturers’ instructions. In this process, 22.5 g of polymer powder was mixed with 10 mL monomer liquid and left for 8–10 min at room temperature in a closed mixing cup. Then, it was pressed into custom plaster molds (8 × 2 mm discs) inside clamp-fixed flasks with a load of 80 bar of pressure. Polymerization was conducted using heat and a cold-water bath that was heated up to 100 °C and left to boil for 45 min. After heat-polymerization, a 30-min room temperature bench-cooling cycle was completed, followed by complete cooling with cold water. Finishing was performed using a silicon carbide paper grit P1200 (Paper SiC P1200; Struers GmbH, Willich, Germany), according to ISO 20795-1 [35]. Confirmation of the final dimensions of each specimen was achieved using a digital caliper. Finally, 24 h before test conduction, specimens were stored in 37 °C water [34,35]. The specimens were then divided into six groups, as follows (N = 60):

(1) ProBase Hot control; “Control HP” (n = 10);
(2) ProBase Hot + 1.5% MPC; “1.5% MPC HP” (n = 10);
(3) ProBase Hot + 3% MPC; “3% MPC HP” (n = 10);
(4) ProBase Hot + 1.5% DMAHDM; “1.5% DMAHDM HP” (n = 10);
(5) ProBase Hot + 3% DMAHDM; “3% DMAHDM HP” (n = 10);
(6) ProBase Hot + 3% MPC + 1.5% DMAHDM; “3% MPC + 1.5% DMAHDM HP” (n = 10).

#### 2.2.2. Three-Dimensionally Printed (3DP) Denture Base Material

The specimens were designed as 8 × 2 mm discs and the design was saved as a standard tessellation language (STL) file. After manual shaking of the resin bottles for 5 min, the contents were then mixed in a mixer (LC-3DMixer; NextDent, Soesterberg, The Netherlands) for 2.5 h, and then poured into the printing tray. The resin was stirred for 30 s inside the tray using a plastic scraper. Printing was carried out via a 3D printing machine (DentalFab; Microlay Dental 3D Printers, Madrid, Spain). The printing settings were set to a 45-degree angle along with the addition of supporting structures. After printing, the specimens were cleaned for 5 min with 90% isopropyl alcohol and then subjected to light-polymerization from all sides for 30 min using a UV-A, type 3, post-polymerization lightbox (LC3DPrint Box; NextDent, Soesterberg, The Netherlands) with an ultraviolet light (385 nm). Finishing was achieved using a silicon carbide paper, grit P1200 (Paper SiC P1200; Struers GmbH, Willich, Germany), according to ISO 20795-1. Confirmation of the final dimensions of each specimen was achieved using a digital caliper. Finally, 24 h before test conduction, the specimens were stored in 37 °C water [34,35]. The specimens were then divided into six groups, as follows (N = 60):

(1) NextDent Denture 3D + control; “Control 3DP” (n = 10);
(2) NextDent Denture 3D + 1.5% MPC; “1.5% MPC 3DP” (n = 10);
(3) NextDent Denture 3D + 3% MPC; “3% MPC 3DP” (n = 10);
(4) NextDent Denture 3D + 1.5% DMAHDM; “1.5% DMAHDM 3DP” (n = 10);
(5) NextDent Denture 3D + 3% DMAHDM; “3% DMAHDM 3DP” (n = 10);
(6) NextDent Denture 3D + 3% MPC + 1.5% DMAHDM; “3% MPC + 1.5% DMAHDM 3DP” (n = 10).

### 2.3. Sample Size

The sample size was digitally calculated with the power package in R software (R package, v1.3-0; R Foundation for Statistical Computing, Vienna, Austria). Eight samples per group were recommended for the detection of a 0.55 effect size (f) and a power of 0.80 at α = 0.05. An increase to 10 samples per group was made to compensate for any specimen damage or loss during the experiments. This modification led to an effect size detection of 0.5 (f) and a power of 0.82 instead [34].

### 2.4. Randomization and Blinding

Each specimen was assigned a random number from 1 to 60 using randomization software (Research Randomizer, V4.0; Social Psychology Network, Middletown, CT, USA) [36] and then sequentially arranged. The randomization codes were revealed after the completion of all tests [34].

### 2.5. Candida albicans CFU Counts

Disc-shaped specimens measuring 8 × 2 mm were disinfected by subjecting them to UV light for 30 min. Sabouraud dextrose broth (Sabrouraud Dextrose Broth; MOLEQULE-ON, New Lynn, Auckland, New Zealand) was prepared following the manufacturer’s instructions and was used as the growth medium. Similarly, Sabouraud dextrose agar (Sabouraud Dextrose Agar; MOLEQULE-ON, New Lynn, Auckland, New Zealand) was prepared following the manufacturer’s instructions and poured into sterile Petri dishes to use as culture plates. A pure culture plate was prepared by picking 10 μL of *C. albicans* colony from an old culture plate with a sterile 10 μL inoculation loop, then striking the freshly prepared agar plate, which was then incubated for 48 h at 37 °C. After incubation, the broth culture was prepared by adding 10 μL of the *Candida albicans* colony from the culture plate into 3 mL of the broth, which was then incubated for 48 h at 37 °C. The same stock culture was used for all samples. After that, the broth culture was visually inspected for turbidity at the bottom of the tube, and then vortexed to ensure proper mixing; this was used as a stock culture. In a 24-well plate, a specimen was placed with 25 μL of the stock culture and 1 mL of the broth into each well, then incubated at 37 °C for 24 h. Subsequently, the specimens were transferred to a new 24-well plate containing 1 mL of fresh broth media and incubated for an additional 24 h to facilitate the development of a relatively mature *Candida albicans* biofilm on the specimens [30]. Harvesting of the biofilms on each specimen was achieved by probe sonication (Fisherbrand™ 505 Sonicator with Probe, ThermoFisher Scientific, Waltham, MA, USA) for 5 min at 20 kHz [37] and vortexing (CorningTM LSETM Vortex Mixer; ThermoFisher Scientific) in a phosphate-buffered solution (PBS; Gibco, Miami, FL, USA), which was then serially diluted into six dilutions. Three 10 μL drops from each dilution were dispersed onto previously prepared agar plates. All plates for all samples were incubated at 37 °C for 48 h, after which the number of colonies was counted on the same day using a CFU plate reader (Reichert Colony Counter, 220V Quebec Darkfield Manual Colony Counter; Reichert Analytical Instruments, Buffalo, NY, USA). The number of colonies and their dilution factor were used to calculate the CFU counts [30].

### 2.6. Cytotoxicity MTT Test

The ISO 10993-5:2009 [38] was followed according to which human periodontal ligament fibroblast was used. The Dulbecco’s modified Eagle medium (DMEM+) (Dulbecco’s modified Eagle medium; MOLEQULE-ON, New Lynn, Auckland, New Zealand) was prepared according to the following protocol: for 50 mL of DMEM+, 44mL of DMEM− was added to a 50 mL sterile centrifuge tube, along with 5 mL of 10% fetal bovine serum, 500 μL of MEM non-essential amino acids, and 500 μL of Pen-strep solution and mixed thoroughly.

The 8 × 2 mm disc-shaped specimens were immersed in 80% ethanol solution and left under UV light for 30 min for disinfection. Subsequently, the specimens were placed in a 48-well plate, and 500 μL of DMEM was added to each well. The plates were then incubated for 24 h, 3 days, and 7 days. Fresh DMEM+ medium was used for each time variable, resulting in three testing media: 24-h testing media, 3-day testing media, and 7-day testing media.

The diluted cell suspension was used to seed the cells into 96-well plates, where 100 μL was added to each well and then incubated at 37 °C with 5% CO_2_ until 80–90% confluency was detected under the microscope. After the intended confluency was reached, the media from each well was discarded and 200 μL of the test media (24 h, 3 days, and 7 days) was added. The well plate was then incubated at 37 °C with 5% CO_2_ until 80–90% confluency of the control cells was detected under the microscope. An MTT assay test (MTT Assay Kit; MOLEQULE-ON, New Lynn, Auckland, New Zealand) was then carried out to assess the cytotoxicity of the tested materials, used according to the manufacturer’s protocol. All media was discarded from each well, after which 90 μL of test media was added along with 10 μL of the MTT solution, then left to continue culturing for 4 h in a dark place away from any light sources. The addition of 110 μL of the formazan dissolving solution in each well was performed and the plate was then placed on a slowly stirring gyratory shaker for 10 min. The plate was then inserted into a microplate reader, which was set at 37 °C with an absorbance of 420 nm.

### 2.7. Statistical Analysis

Statistical analysis was performed with SPSS Statistics v.20 (IBM, Endicott, Armonk, NY, USA) at α = 0.05. Two independent variables were assessed: the two types of acrylic materials, and the DMAHDM- and/or MPC-incorporated acrylic materials. A multivariant analysis of variance (MANOVA) test was performed to compare the mean differences between the groups.

## 3. Results

### 3.1. Candida albicans CFU Counts

**Figure 1:**
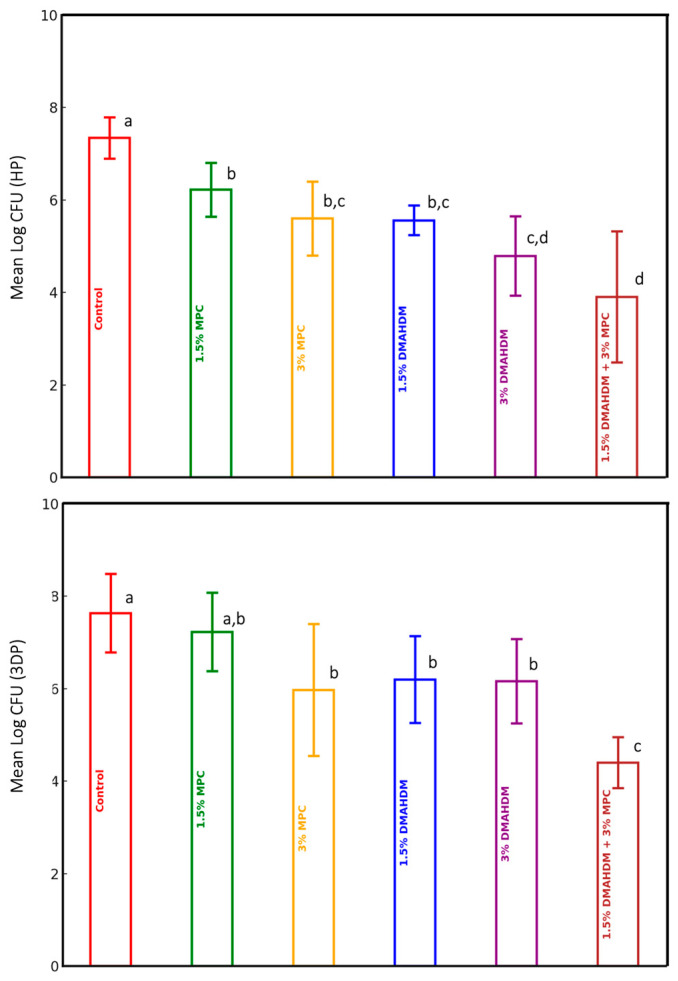
Log CFU count of the HP and 3DP materials (mean ± SD). Dissimilar letters indicate values that are significantly different from each other (*p* ≤ 0.05). Error bars inside the columns represents the standard deviation.

The comparison of mean log CFU values among the experimental groups for the study materials (HP and 3DP) is presented in Table 1 and Figure 1. A statistically significant difference in the mean log CFU values was observed among the experimental groups in both studied materials (*p* < 0.0001). For the HP material, the incorporation of DMAHDM and MPC, either individually or in combination, significantly reduced the CFU count compared to the control group, which lacked DMAHDM and MPC (*p* ≤ 0.05). The mutual addition of 1.5% DMAHDM and 3% MPC resulted in the highest CFU log reduction of approximately 3.5-fold when compared to the control group (*p* ≤ 0.05). A significant difference was also detected between one group (1.5% MPC) and other groups ((3% DMAHDM) and (1.5% DMAHDM and 3% MPC)), wherein the prior group showed higher CFU counts. The latter group (1.5% DMAHDM and 3% MPC) also revealed significantly lower CFU counts than the others (3% MPC) and (1.5% DMAHDM) (Table 1 and Figure 1).

Regarding the 3DP material, the incorporation of DMAHDM and 3% MPC, either individually or in combination, resulted in a significant reduction in the CFU count when compared to the control group (*p* ≤ 0.05). Similarly, the mutual addition of 1.5% DMAHDM and 3% MPC resulted in the highest CFU log reduction, of approximately threefold, compared to the control group (*p* ≤ 0.05), which also significantly outperformed all the remaining experimental groups (Table 1 and Figure 1).

### 3.2. Cytotoxicity MTT Test

**Figure 2:**
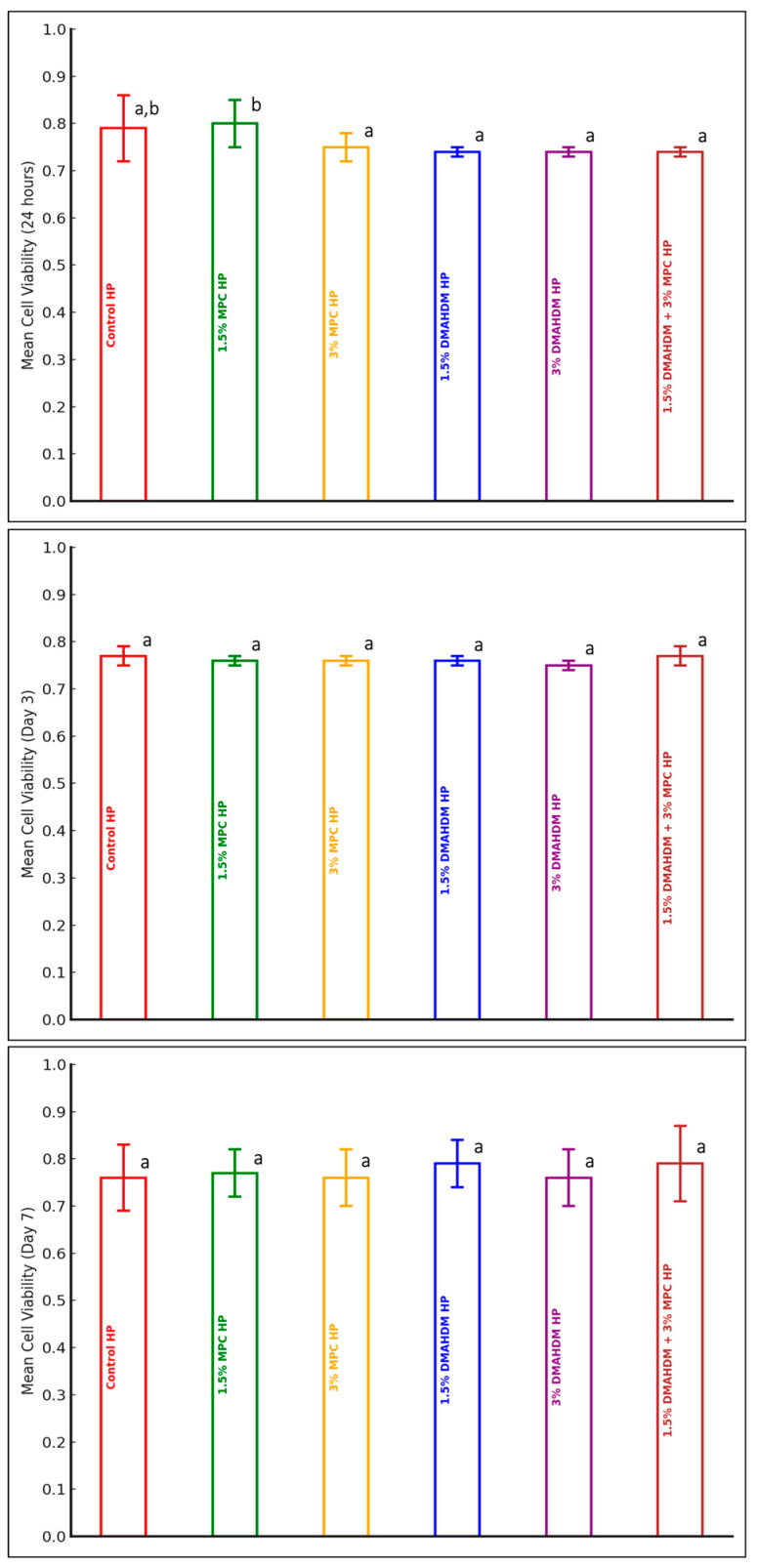
Cell viability of the HP material (mean ± SD) at three observation time points: 24 h, Day 3, and Day 7. Dissimilar letters indicate values that are significantly different from each other (*p* ≤ 0.05). Error bars inside the columns represents the standard deviation.

A comparison of the mean cell viability values among the experimental groups of the HP material at each of the three time points (24 h, Day 3, and Day 7) is presented in Table 2 and Figure 2. A statistically significant difference in the mean cell viability values among the experimental groups of the HP material was only detected at 24 h (*p* = 0.001). Only the addition of 1.5% MPC had a statistically significant higher cell viability value in comparison to the other groups. However, all groups, at all time points, maintained a >70% cell viability percentage, which indicates the biocompatibility of the modified HP material (Table 2 and Figure 2).

**Figure 3:**
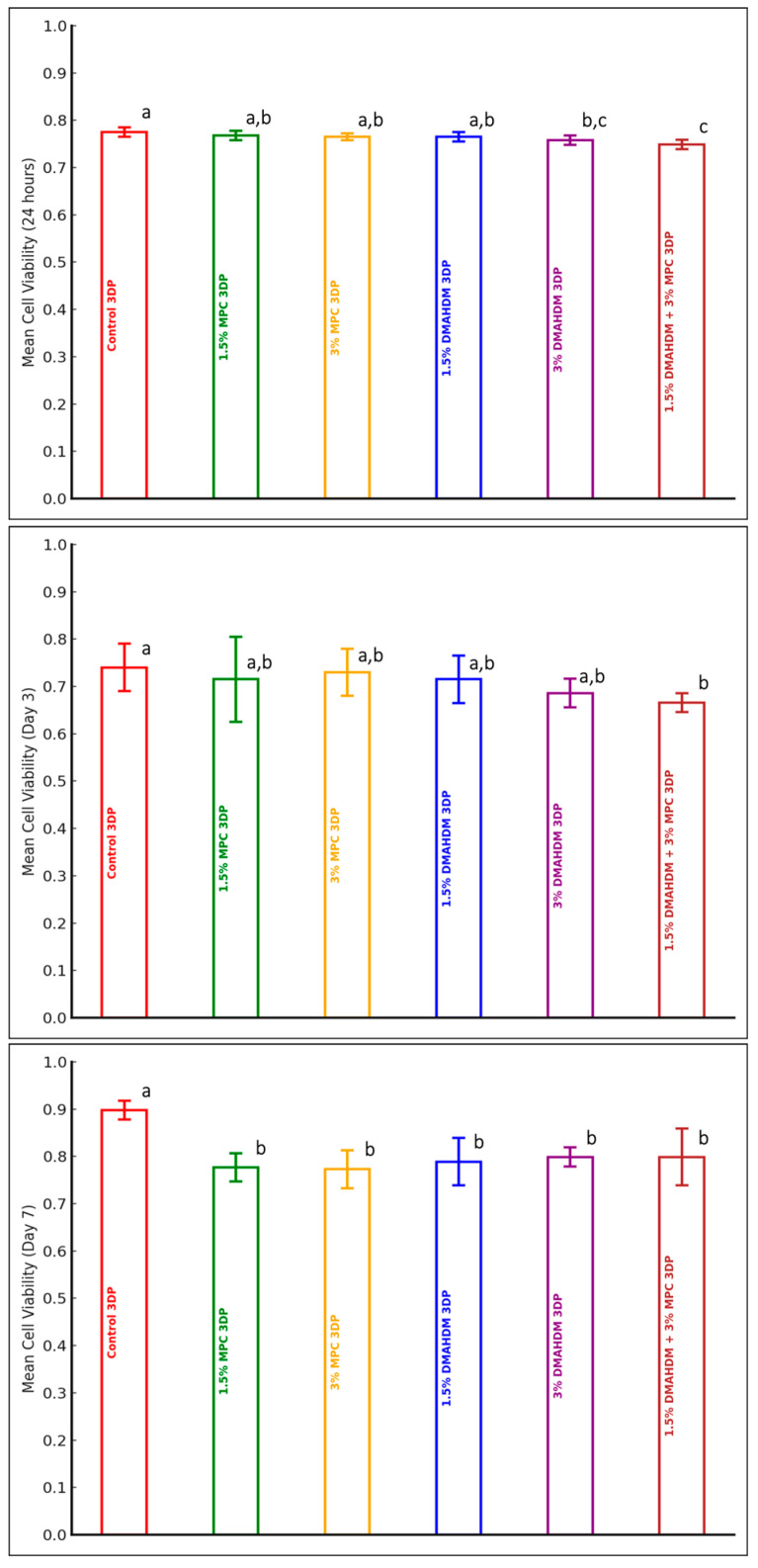
Cell viability of 3DP material (mean ± SD) at three observation time points: 24 h, Day 3, and Day 7. Dissimilar letters indicate values that are significantly different from each other (*p* ≤ 0.05). Error bars inside the columns represents the standard deviation.

A comparison of the mean cell viability values among the experimental groups of the 3DP material at each of the three time points (24 h, Day 3, and Day 7) is presented in Table 3 and Figure 3. A statistically significant difference in the mean cell viability values among the experimental groups was detected after 24 h (*p* < 0.0001), on Day 3 (*p* = 0.043), and on Day 7 (*p* < 0.0001). The addition of 3% DMAHDM or a combination of 1.5% DMAHDM and 3% MPC evinced statistically significant lower cell viability in comparison to the control group at 24 h. The combination of 1.5% DMAHDM and 3% MPC evinced significantly lower cell viability in comparison to groups containing MPC alone or 1.5% DMAHDM at 24 h. On Day 3, only the addition of DMAHDM and MPC together presented a significantly lower cell viability value in comparison to the control group. On Day 7, the addition of DMAHDM and MPC, alone or together, evinced significantly lower cell viability values in comparison to the control group. However, all groups, at all time points, maintained a >70% cell viability percentage, which indicates biocompatibility (Table 3 and Figure 3).

## 4. Discussion

This study examined the effect of incorporating dimethylaminohexadecyl methacrylate (DMAHDM) and 2-methacryloyloxyethyl phosphorylcholine (MPC) into heat-polymerized (HP) and 3D-printed (3DP) denture base resins and their impact on microbial adhesion and cytotoxicity.

The incorporation of DMAHDM and MPC significantly reduced the microbial adhesion of *Candida albicans*, which is consistent with findings from previous studies. For instance, Bajunaid et al. demonstrated that incorporating 1.5% DMAHDM and 3% MPC into PMMA significantly reduced *C. albicans* biofilm formation by twofold compared with control groups that were devoid of these agents [31]. These findings are in accordance with the current study’s results, wherein the combination of DMAHDM and MPC showed the most substantial decrease in CFU counts for both HP and 3DP materials. Moreover, Al-Dulaijan and Balhaddad reported that bioactive denture base resins containing quaternary ammonium compounds like DMAHDM effectively suppressed the growth of denture stomatitis-related pathogens, including *C. albicans*, by disrupting the biofilm matrix and reducing its microbial adhesion [39]. This finding aligns with the present results, confirming the antimicrobial efficacy of DMAHDM and MPC in denture base materials. Notably, the combination of 1.5% DMAHDM and 3% MPC exhibited the most substantial decrease in CFU counts for both HP and 3DP materials. The significant reduction in CFU counts with higher concentrations of DMAHDM (3%) suggests a dose-dependent antimicrobial effect, corroborating the findings of Li et al. and Zhou et al. on the antibacterial properties of quaternary ammonium compounds [21,22]. The MTT assay is a colorimetric method used to assess cell metabolic activity, serving as an indicator of cell viability, proliferation, and cytotoxicity. The key to interpreting MTT assay results lies in calculating the percentage cell viability by comparing the absorbance (optical density) of treated cells to that of untreated control cells, using the following formula:

$$\mathrm{Percent}\;\mathrm{Cell}\;\mathrm{Viability}=(\frac{\mathrm{Absorbance}\;\mathrm{of}\;\mathrm{Control}\;\mathrm{Cells}}{\mathrm{Absorbance}\;\mathrm{of}\;\mathrm{Treated}\;\mathrm{Cells}\;})\;\times \;100$$

A percentage cell viability of less than 70% is often considered to indicate significant cytotoxicity. Some studies may use other criteria, such as a percentage cell viability of less than 50% to indicate strong cytotoxic effects. In summary, an MTT assay value is typically considered to indicate cytotoxicity when the viability of the treated cells falls below 70% compared to the control cells. This threshold can vary, depending on the specific context and criteria established by a given study or experiment [40,41,42].

In the present study, the cytotoxicity results varied between HP and 3DP materials. At 24 h, neither of the materials showed statistically significant differences among groups in comparison with the control group. This trend continued on Day 3 and Day 7 for the HP material. However, for the 3DP material, Day 7 revealed a highly statistically significant difference between the control group in comparison to the rest of the groups. This might seem to indicate the cytotoxicity of the other groups, but when we return to the cytotoxicity threshold of 70% cell viability, we can see that even though the difference is statistically significant, the other groups maintained a percentage cell viability of >70%, which excludes significant cytotoxicity. Moreover, the combination of 1.5% DMAHDM + 3% MPC consistently showed lower percentage cell viability values, but it also maintained a >70% value excluding significant cytotoxicity. These findings are consistent with other studies indicating that DMAHDM and MPC can reduce microbial adhesion without significantly compromising biocompatibility [31,43,44].

Incorporating DMAHDM and/or MPC into denture base materials shows promising clinical potential for preventing denture-related infections, such as denture stomatitis, which is commonly linked to *Candida albicans* biofilms. The observed antimicrobial efficacy, coupled with the acceptable cytotoxicity levels, suggest that these modified materials could enhance oral health outcomes for denture wearers. Future research should prioritize long-term clinical trials to validate these findings and explore the durability and mechanical properties of these modified denture bases under clinical conditions. Additionally, investigating broader microbial testing would improve generalizability. Finally, testing the effects of these antimicrobial agents on other microbial species and biofilms could further establish their broad-spectrum applicability in dental materials [39,43].

## 5. Conclusions

Based on the findings of this in vitro study, the following conclusions can be made:

1. The incorporation of DMAHDM and/or MPC into 3DP is a novel approach filling a gap in the current knowledge.
2. The incorporation of DMAHDM and/or MPC into HP and 3DP denture base resin materials significantly reduces microbial adhesion.
3. The incorporation of DMAHDM and/or MPC into HP and 3DP denture base resin materials maintains acceptable cytotoxicity levels.
4. The incorporation of DMAHDM and/or MPC into HP and 3DP denture base resin materials offers a promising strategy for improving the antimicrobial properties of the modified denture materials.

## Figures and Tables

**Table 1:** Comparison of the mean log CFU values among the experimental groups of both study materials (HP and 3DP).

Groups	Material					
	HP			3DP		
	Mean Log CFU (SD)	*F*-Value	*p*-Value	Mean Log CFU (SD)	*F*-Value	*p*-Value
Control (n = 10)	7.34 (0.45)	20.693	<0.0001	7.63 (0.85)	13.873	<0.0001
1.5% MPC (n = 10)	6.22 (0.58)			7.23 (0.85)		
3% MPC (n = 10)	5.60 (0.80)			5.97 (1.43)		
1.5% DMAHDM (n = 10)	5.56 (0.32)			6.20 (0.94)		
3% DMAHDM (n = 10)	4.79 (0.86)			6.16 (0.91)		
1.5% DMAHDM + 3% MPC (n = 10)	3.90 (1.42)			4.40 (0.55)		

HP: Heat-polymerized; 3DP: 3D-printed; CFU: colony-forming unit.

**Table 2:** Comparison of mean cell viability values between the experimental study groups of HP material at each of three observation time periods (24 h, Day 3, and Day 7).

Groups	Time Points								
	24 h			Day 3			Day 7		
	Mean Cell Viability (SD)	*F*-Value	*p*-Value	Mean Cell Viability (SD)	*F*-Value	*p*-Value	Mean Cell Viability (SD)	*F*-Value	*p*-Value
Control HP (n = 10)	0.79 (0.07)	4.830	0.001	0.77 (0.02)	1.955	0.102	0.76 (0.07)	0.430	0.826
1.5% MPC HP (n = 10)	0.80 (0.05)			0.76 (0.01)			0.77 (0.05)		
3% MPC HP (n = 10)	0.75 (0.03)			0.76 (0.01)			0.76 (0.06)		
1.5% DMAHDM HP (n = 10)	0.74 (0.01)			0.76 (0.01)			0.79 (0.05)		
3% DMAHDM HP (n = 10)	0.74 (0.01)			0.75 (0.01)			0.76 (0.06)		
1.5% DMAHDM + 3% MPC HP (n = 10)	0.74 (0.01)			0.77 (0.02)			0.79 (0.08)		

HP: Heat-polymerized.

**Table 3:** Comparison of mean cell viability values between the experimental study groups of 3DP material at each of three observation time periods (24 h, Day 3, and Day 7).

Groups	Time Points								
	24 h			Day 3			Day 7		
	Mean Cell Viability (SD)	*F*-Value	*p*-Value	Mean Cell Viability (SD)	*F*-Value	*p*-Value	Mean Cell Viability (SD)	*F*-Value	*p*-Value
Control 3DP (n = 10)	0.775 (0.01)	7.29	< 0.0001	0.740 (0.05)	2.49	0.043	0.898 (0.02)	10.37	< 0.0001
1.5% MPC 3DP (n = 10)	0.768 (0.01)			0.715 (0.09)			0.777 (0.03)		
3% MPC 3DP (n = 10)	0.765 (0.007)			0.730 (0.05)			0.773 (0.04)		
1.5% DMAHDM 3DP (n = 10)	0.765 (0.01)			0.715 (0.05)			0.789 (0.05)		
3% DMAHDM 3DP (n = 10)	0.758 (0.01)			0.686 (0.03)			0.799 (0.02)		
1.5% DMAHDM + 3% MPC 3DP (n = 10)	0.749 (0.01)			0.666 (0.02)			0.799 (0.06)		

3DP: 3D-printed.

## Data Availability

The original contributions presented in the study are included in the article, further inquiries can be directed to the corresponding author.
